# Susceptibility and Synergistic Effects of Guava Plant Extract and Antimicrobial Drugs on Escherichia coli

**DOI:** 10.7759/cureus.52345

**Published:** 2024-01-15

**Authors:** Sohini Mitra, Anahita V Bhesania Hodiwala, Harapriya Kar

**Affiliations:** 1 Department of Microbiology, MGM (Mahatma Gandhi Mission) Medical College and Hospital, Navi Mumbai, IND

**Keywords:** urinary tract infection, synergistic activity, antimicrobial activity, aqueous extract, escherichia coli, psidium guajava

## Abstract

Introduction

*Psidium guajava* (guava) is a fruit plant of the Myrtaceae family. Guava roots, leaves, and fruits have traditionally been used to prevent and treat various infections. In the last few decades, there has been exponential growth in herbal medicine. Therefore, the present study was conducted to determine the susceptibility and synergistic properties of the antimicrobial activity of the aqueous leaf extract of guava and other antimicrobial drugs against *Escherichia coli* (*E. coli*).

Methodology

A prospective observational study was conducted at the Department of Microbiology, MGM Medical College and Hospital, Navi Mumbai, India, involving 180 urine samples collected from patients who exhibited symptoms of urinary tract infection (UTI). The aim was to evaluate in vitro synergism between leaf extracts of guava and antimicrobial drugs on uropathogenic *E. coli*, using minimal inhibitory concentration (MIC) and the Kirby-Bauer method. The Kirby-Bauer disc diffusion method was employed to determine the synergistic activity using Muller-Hinton agar (MHA), and the zone of inhibition was measured in millimeters.

Results

The study found that, of the 180 urine samples collected from patients with UTI, significant growth was observed in 93 samples, with the most notable increase seen in *E. coli*. The antibiotics tobramycin, ofloxacin, and amikacin, each showing a sensitivity of 76% and 70% respectively, were found to be the most sensitive. Conversely, cefuroxime and cephalothin, both at 76%, were the most resistant. Furthermore, the antibiotic sensitivity pattern of *E. coli* without guava extract demonstrated tobramycin (TOB) at 76.66%, followed by ofloxacin (OF) and amikacin (AK) at 70% each, levofloxacin (LE) at 63.33%, nitrofurantoin (NIT) at 53.33%, trimethoprim (TR) at 43.33%, cefotaxime (CTX) at 36.66%, ceftizoxime (CZX) at 30%, norfloxacin (NR) at 26.66%, cephalothin (CEP) at 23.33%, amoxicillin-clavulanate (AMC) at 20%, and cefuroxime (CXM) at 10%. In contrast, when the antibiotic sensitivity pattern of E. coli with guava extract was examined, the highest sensitivity was noted for OF (100%), followed by LE (96.66%), TOB (93.33%), AK (90%), NIT (76.66%), AMC and TR (66.66% each), CTX (60%), CZX (53.33%), CEP (50%), NX (43.33%), and CXM (26.66%). Therefore, *Psidium guajava* (guava) extract exhibited a synergistic effect when combined with antibiotics, most notably with ofloxacin.

Conclusion

The study revealed that the highest synergistic activity of guava plant leaf extract was with the antibiotic ofloxacin. This finding indicates that guava extract enhances the effectiveness of commonly used antibiotics for treating UTI, an effect mainly attributed to the flavonoid compounds and their derivatives in the guava leaf extract, which inhibit bacterial growth. This study demonstrated the antibacterial properties of guava, suggesting that combining antibiotics with guava extract can help delay the emergence of bacterial resistance.

## Introduction

*Escherichia coli* (*E. coli*) is one of the most important species encountered clinically and has been associated with various infections such as urinary tract infection (UTI), diarrhea, and meningitis [1]. Plants have long been valuable sources of natural compounds for sustaining human health, with more extensive investigations for natural remedies. The World Health Organization recognizes medicinal plants as the finest source of a wide range of medications [2]. Approximately 80% of people in developed nations utilize plants as traditional medicine [2]. As a result, such plants should be researched to better understand their characteristics, safety features, and efficiency [2]. Multiple drug resistance (MDR) has emerged in human pathogenic bacteria as a result of the widespread application of commercial antimicrobial medications frequently in the treatment of infectious diseases. These circumstances prompted scientists to look for new antimicrobial compounds from other sources as innovative antimicrobial chemotherapeutic agents [3].

*Psidium guajava*, known as apple guava, has shown a range of biological activities linked to its main constituents, which include flavonoids, phenolic compounds, carotenoids, and terpenoids. It is a plant used in traditional medicine that has phytotherapeutic properties. *Psidium guajava* phytochemical studies on tannins, flavonoids, essential oils, and proteins have been documented. This plant also has hypoglycemic antibacterial and antimutagenic characteristics [4]. Synergism is defined as the conjugation of discrete agents (such as medications) or instances in such a way that the cumulative effect exceeds the sum of the distinct effects [5]. Hence, this study aimed to evaluate in vitro synergism between leaf extracts of guava (by the maceration method) and antimicrobial drugs against uropathogenic *E. coli* from UTI by using the minimal inhibitory concentration (MIC) and Kirby-Bauer disc diffusion methods.

## Materials and methods

A prospective observational study was conducted in the Department of Microbiology at MGM Medical College and Hospital, Navi Mumbai, India, following approval from the Institutional Ethics Committee (IEC) with reference number N-EC/2022/02/10. A total of 180 urine samples were screened for UTI, among which 93 samples were culture-positive. Of these, 30 samples were *E. coli *culture-positive, and after obtaining written informed consent, the samples were included in the study. Pathogenic strains of *E. coli* were obtained from urine samples and isolated on MacConkey agar. The powdered leaf extract of *Psidium guajava* (50 g) was mixed with 250 ml of distilled water in a conical flask and kept covered for 48 hours in a dark place at room temperature using the Soxhlet apparatus extraction method [6,7].

The MIC is the lowest concentration of an antimicrobial that suppresses bacterial growth. A broth dilution procedure was employed to determine the MIC of the plant extract. The extracts were serially diluted twofold in Mueller-Hinton broth. The guava extract showed an MIC of 25 mg/ml against uropathogenic *E. coli* from stock concentrations of 200 mg/ml, resulting in 100, 50, 25, and 12.5 mg/ml of the extracts in the broth, respectively. Hence, 0.1 ml of the 25 mg/ml concentration was used to prepare filter paper discs (antibiotic and guava extract) for further testing. The turbidity of the cultures was maintained according to the 0.5 McFarland standard, and the inoculum size was 1 X 10^8 cells [8,9].

The disk diffusion method developed by Kirby and Bauer was utilized for antibiotic susceptibility testing. Following the guidelines of the Clinical Laboratory Standard Institute (CLSI) (2022), Mueller-Hinton agar (MHA) was used for inoculation, and antibiotic discs (Himedia) available for purchase were used. Inoculums were prepared by transferring a single colony with a sterile nichrome loop to 4 ml of peptone water and incubated at 37°C for 2-4 hours to achieve turbidity comparable to a 0.5 McFarland suspension. A sterile cotton swab was then used to seed the MHA plate with the suspension by lawning the entire surface of the media. After lawning, one side of the plates was covered with an antibiotic disc from Himedia using forceps, while the other side was covered with commercially available antimicrobial discs. Subsequently, 20 µl of guava extract was cautiously applied onto the antibiotic disc. The plates were incubated for 24 hours at 37°C, and the zone of inhibition was then measured in mm using a transparent ruler [9-11]. The antibiotics used included ofloxacin (OF) 2 mcg, cephalothin (CEP) 30 mcg, levofloxacin (LE) 5 mcg, ceftizoxime (CZX) 30 mcg, nitrofurantoin (NIT) 30 mcg, cefuroxime (CXM) 30 mcg, trimethoprim (TR) 5 mcg, cefotaxime (CTX) 30 mcg, amoxicillin-clavulanate (AMC) 30 mcg, norfloxacin (NX) 10 mcg, amikacin (AK) 30 mcg, and tobramycin (TOB) 10 mcg.

## Results

Overall, 180 urine samples from patients exhibiting symptoms of UTI were screened, among which 93 (51.66%) samples were microbiologically confirmed cases, as they showed significant growth. Of these, 30 samples were E. coli culture-positive UTI samples. The distribution of organisms isolated from the urine samples of the 93 UTI patients is described in Table 1.

**Table 1:** Organisms isolated from urine samples.

Organisms	Numbers	Percentage (%)
Escherichia coli	30	32.25
Enterococcus	9	9.67
Klebsiella pneumoniae	6	6.52
Citrobacter	13	13.97
Proteus mirabilis	3	3.22
Candida species / albicans	17	18.27
Staphylococcus aureus	11	11.82
Acinetobacter	4	4.30

The antibiotic sensitivity pattern of *E. coli* is demonstrated in Table 2.

**Table 2:** Antibiotic sensitivity pattern in 30 patients showing growth of E. coli. *E. coli* = *Escherichia coli*, CEP = cephalothin, OF = ofloxacin, CZX = ceftizoxime, LE = levofloxacin, CXM = cefuroxime, NIT = nitrofurantoin, CTX = cefotaxime, TR = trimethoprim, AMC = amoxicillin-clavulanate, and AK = amikacin, NX = norfloxacin, and TOB = tobramycin

Patients	CEP	OF	CZX	LE	CXM	NIT	CTX	TR	AMC	AK	NX	TOB
1	_---	12	---	22	24	16	---	16	---	12	15	---
2	---	17	---	21	---	12	---	---	14	11	---	14
3	5	14	22	14	---	16	14	10	7	15	---	15
4	---	18	4	17	---	7	---	---	10	16	20	16
5	6	13	---	16	---	19	15	8	19	17	12	15
6	---	16	---	17	---	19	16	3	13	17	05	15
7	---	17	20	22	---	21	14	7	18	18	---	16
8	---	12	21	14	16	19	18	12	18	18	10	14
9	---	16	---	---	21	---	---	---	10	---	---	17
10	---	15	---	23	---	---	---	---	7	---	---	18
11	---	8	12	22	---	---	---	8	13	18	---	12
12	---	13	---	22	26	19	---	---	18	19	---	---
13	7	26	27	33	---	19	---	24	---	24	28	21
14	---	16	---	20	---	21	---	18	12	22	---	20
15	29	17	---	21	---	19	22	06	---	23	---	19
16	22	26	28	28	---	---	26	23	16	20	---	23
17	---	19	25	19	22	---	25	19	17	26	27	22
18	26	23	26	24	10	18	27	25	07	05	23	12
19	---	19	---	22	---	---	22	13	14	11	---	13
20	27	30	33	31	---	21	31	16	---	25	27	21
21	---	20	---	20	7	23	---	25	5	23	---	19
22	---	16	---	20	---	20	19	27	---	22	---	21
23	---	20	22	14	---	23	08	11	---	23	---	24
24	---	26	29	34	14	18	---	25	13	24	12	25
25	27	30	33	31	8	21	31	27	---	25	28	21
26	---	12	27	24	18	23	12	---	08	23	---	25
27	---	18	23	28	---	---	10	19	---	12	17	18
28	24	28	23	30	---	21	31	27	25	28	08	20
29	---	14	18	27	24	15	05	---	19	22	---	21
30	22	25	27	29	---	18	28	---	---	22	27	16
Sensitive	07	21	09	19	03	16	11	13	06	21	08	23
%	23.3	70	30	63.3	10	53.3	36.6	43.3	20	70	26.6	76.6
Intermediate	00	05	04	06	04	05	02	03	04	02	01	03
%	00	16.6	13.3	20	13.3	16.6	6.66	10	13.3	6.66	33.3	10
Resistant	23	04	17	05	23	09	17	14	20	07	21	04
%	76.6	13.3	56.6	16.6	76.6	30	56.6	46.6	66.66	23.3	70	13.3

**Figure 1:**
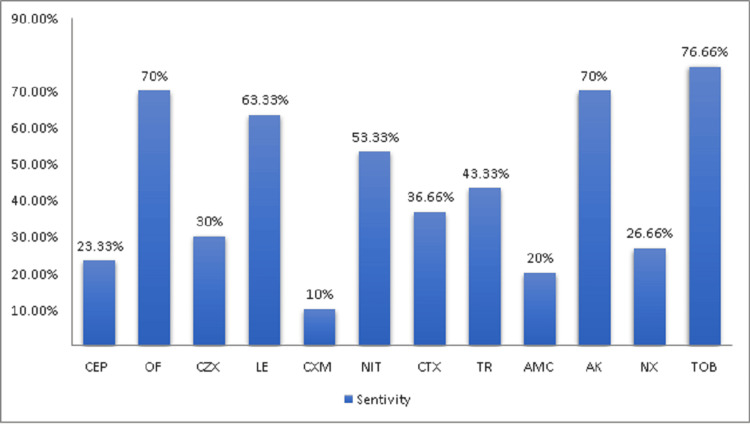
Sensitivity pattern on E. coli without guava extract. *E. coli *= *Escherichia coli*, CEP = cephalothin, OF = ofloxacin, CZX = ceftizoxime, LE = levofloxacin, CXM = cefuroxime, NIT = nitrofurantoin, CTX = cefotaxime, TR = trimethoprim, AMC = amoxicillin-clavulanate, and AK = amikacin, NX = norfloxacin, and TOB = tobramycin

The antibiotic sensitivity pattern of *E. coli *without guava extract is illustrated in Figure 1.

**Figure 2:**
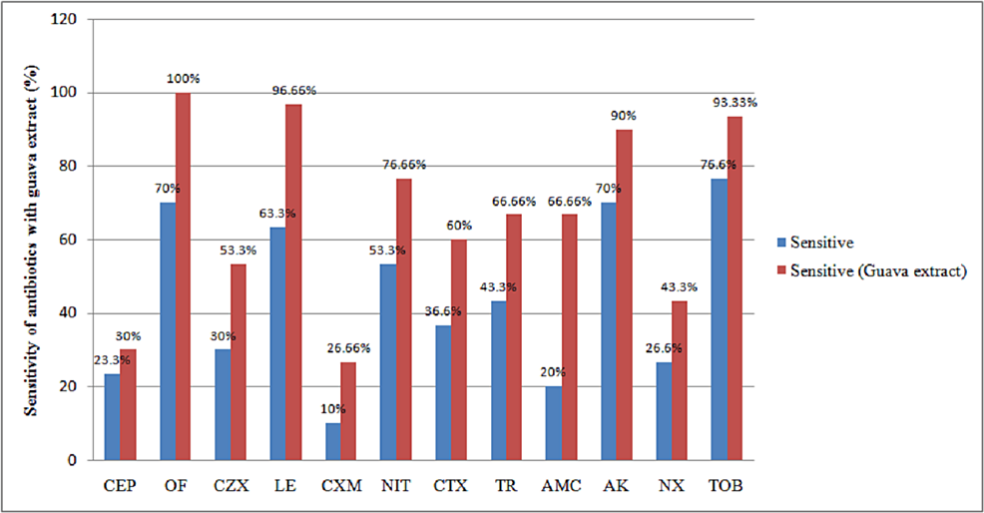
Synergistic activity of antibiotics with guava extract. CEP = cephalothin, OF = ofloxacin, CZX = ceftizoxime, LE = levofloxacin, CXM = cefuroxime, NIT = nitrofurantoin, CTX = cefotaxime, TR = trimethoprim, AMC = amoxicillin-clavulanate, and AK = amikacin, NX = norfloxacin, and TOB = tobramycin.

The synergistic activity of antibiotics with guava extract is depicted in Figure 2.

**Figure 3:**
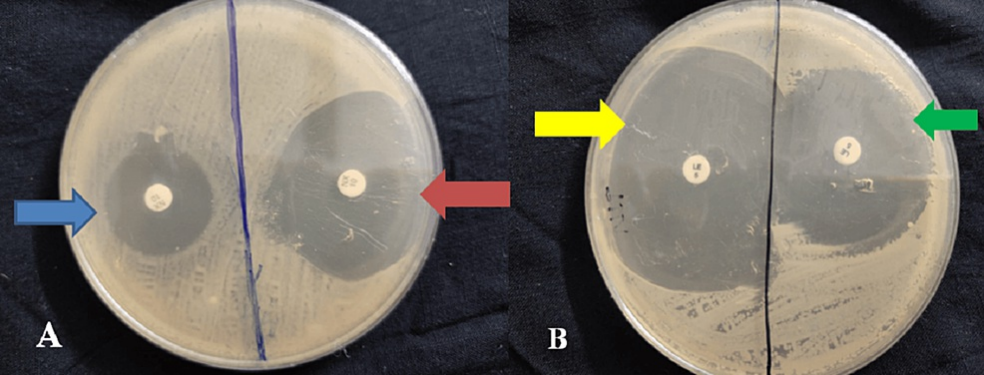
Specimen illustrating sensitivity without guava extract and synergistic activity with guava extract. A: The blue arrow illustrates sensitivity without guava extract, and the red arrow illustrates sensitivity with guava extract. B: The yellow arrow illustrates sensitivity with guava extract and the green arrow illustrates sensitivity without guava extract.

The specimen illustrating sensitivity without guava extract and synergistic activity with guava extract is depicted in Figure 3.

## Discussion

The current study included 180 urine samples from UTI patients, out of which growth of organisms was observed in a total of 93 samples. The most significant growth was of the organism *E. coli*, found in 30 samples (32.25%), followed by *Candida albicans *in 17 samples (18.27%), *Citrobacter* in 13 samples (13.97%), *Staphylococcus aureus* in 11 samples (11.82%), *Enterococcus* in nine samples (9.67%), *Klebsiella pneumoniae* in six samples (6.52%), *Acinetobacter* in four samples (4.30%), and *Proteus mirabilis* in three samples (3.22%). In a similar study of 351 samples, 316 showed positive culture growth, with 64% being *Escherichia coli*, 6.32% *Klebsiella pneumoniae*, 10.7% *Enterococcus* species, 3.16% *Pseudomonas aeruginosa*, 4.43% *Candida albicans*, 5.06% *Staphylococcus aureus*, and 5.69% other microorganisms such as *Acinetobacter*, *Staphylococcus epidermidis*, and *Citrobacter* [12].

Another study conducted by Gul and Gurbuz involved 1211 urine samples, in which the organisms isolated were 0.17% *Acinetobacter*, 0.58% of *Acinetobacter baumannii*, 0.08% of *Alcaligenes faecalis*, 0.08% of *Burkholderia cepacia*, 1.24% of *Candida albicans*, 0.08% of *Candida famata*, 0.17% of *Candida kefyr*, 0.08% of *Candida krusei*, 0.25% of *Candida spherica*, 0.25% of *Candida tropicalis*, 0.17% of *Citrobacter freundii*, 0.25% of *Citrobacter koseri*, 0.08% of *Enterobacter aerogenes*, 0.74% of *Enterobacter cloacae*, 0.33% of *Enterococcus* species, 4.5% of *Enterococcus faecalis*, 0.74% of *Enterococcus faecium*, 68.4% of *Escherichia coli*, 2.8% of *Klebsiella* species, 0.5% of *Klebsiella oxytoca*, 7.2% of *Klebsiella pneumoniae*, 0.25% of *Morganella morganii*, 0.33% of *Proteus* species, 1.73% of *Proteus mirabilis*, 0.33% of *Providencia rettgeri*, 1.2% of *Pseudomonas aeruginosa *10.08% of *Salmonella* species, 0.17% of *Serratia fonticola*, 0.17% of *Serratia liquefaciens*, 0.08% of *Serratia marcescens*, 0.08% of *Shigella sonnei*, 0.33% of *Staphylococcus aureus*, 1.9% of *Staphylococcus epidermidis*, 0.25% of *Staphylococcus haemolyticus*, 0.08% of *Staphylococcus hominis*, 0.5% of *Staphylococcus saprophyticus*, 0.08% of *Staphylococcus warneri*, 0.17% of *Streptococcus* species, 15% of *Streptococcus agalactiae*, 0.08% of *Streptococcus constellatus*, 0.17% of *Streptococcus dysgalactiae*, 0.25% of *Streptococcus mitis*, 0.08% of *Streptococcus salivarius*, and 0.08% of *Streptococcus sanguinis *[13]. In the present study, the incidence of E. coli was 51.66%, which was quite similar to the findings reported by Hamza et al., but lower than the incidence reported by Gul and Gurbuz, who found E. coli in 68.4% of 1211 urine samples [12,13].

Additionally, Sabir et al. in their study reported that *E. coli* were highly resistant to penicillin (100%), amoxicillin (100%), and cefotaxime (89.7%), followed by an intermediate level of resistance to ceftazidime (73.8%), tetracycline (69.4%), cephradine (73.8%), augmentin (62.6%), gentamycin (59.8%), doxycycline (66.6%), ciprofloxacin (54.2%), cefuroxime (58.2%), aztreonam (44.8%), cefaclor (50%), imipenem (43.3%), and ceftriaxone (43.3%), and a low level of resistance to kanamycin (19.9%), streptomycin (30%), amikacin (12.7%), and tazocin (14%), and the lowest to norfloxacin (11.2%). Two hundred sixty-one (81%) of the 321 E. coli isolates were found to be multidrug-resistant, and five (1.5%) were found to be extensively drug-resistant [14]. Furthermore, Ferdosi-Shahandashti's analysis showed that 57 samples of urine contained *E. coli *growth, and the most sensitive antibiotics were ofloxacin, imipenem, and ciprofloxacin, with 87.7%, 87.7%, and 78.9% sensitivity, respectively. The most resistant antibiotics were cefixime, cotrimoxazole, cefotaxime, and ceftriaxone [15].

Shalini et al. carried out a similar study in a tertiary hospital to examine the shifting patterns of antibiotic sensitivity among uropathogens causing UTIs. A total of 170 urine culture sensitivity reports were examined, where *E. coli *predominated. Further antibiotic testing showed amoxicillin with a 19.60% sensitivity and 80.40% resistance, amoxiclav with 27.20% sensitivity and 72.80% resistance, cotrimoxazole with 19.60% sensitivity and 80.40% resistance, gentamicin with 66.30% sensitivity and 43.70% resistance, amikacin with 98.91% sensitivity and 1.09% resistance, ciprofloxacin with 69.56% sensitivity and 30.44% resistance, norfloxacin with 73.91% sensitivity and 26.09% resistance, levofloxacin with 75% sensitivity and 25% resistance, nitrofurantoin with 93.48% sensitivity and 6.52% resistance, and ceftazidime with 80.43% sensitivity. Minocycline showed 75% sensitivity and 25% resistance. The results demonstrated that *E. coli* showed high sensitivity to amikacin, which is 98.91%, nitrofurantoin 93.48%, and ceftazidime 80.43% [16]. In the current study, ofloxacin, tobramycin, and amikacin were reported to be the most sensitive, that is, 70%, 76.6%, and 70%, respectively, and cefuroxime and cephalothin were found to be the most resistant, that is, 76.6% for both.

Additionally, the present study used guava extract to observe the synergistic activity with the combination of 12 antibiotics by the Kirby-Bauer disc diffusion method on MHA and reported that the sensitivity percentage has increased by 7-10% on average, indicating the synergistic activity of *Psidium guajava*. Furthermore, in a study by Dalee et al., the concentration of tetracycline was fixed (6.25 μg/ml) for *E. coli*, and it was observed that synergy was achieved as MIC and minimum bactericidal concentration (MBC) values reduced, regardless of the extracting solvent used, as guava MIC decreased twofold and more, and MBC decreased up to eightfold [17].

The guava leaves are two to six inches long and one to two inches broad. They have noticeable veins, a dull-green appearance, and a coriaceous, stiff texture when crushed. The guava leaf contains bioactive ingredients that have antimicrobial, blood-glucose-regulating, and even weight-loss properties. An essential oil rich in cineol, tannins, triterpenes, flavonoids, resin, eugenol, malic acid, fat, cellulose, chlorophyll, and several other fixed compounds can be found in guava leaves [18,19]. In a study by Biswas et al., the antimicrobial potential of guava (*Psidium guajava*) leaf extracts against two gram-negative bacteria (*E. coli *and *Salmonella enteritidis*) and two gram-positive bacteria (*Staphylococcus aureus *and *Bacillus cereus*) was evaluated. Only two of the crude solvent extracts made from *Psidium guajava *leaves, methanol and ethanol, had inhibitory efficacy against bacteria, according to the study's findings. The two extracts only demonstrated inhibition against the two gram-positive bacteria, *Bacillus cereus *and *Staphylococcus aureus*, while the gram-negative bacteria did not show any inhibition at all. These results contradicted the outcomes of the present study. Additionally, Hoque et al. found no antibacterial activity of ethanolic extracts of guava against *E. coli *and *S. enteritidis *[20]. The outcomes of the above-mentioned studies contradicted the findings of the present study; however, Vieira et al. found guava sprout extracts were effective in inhibiting *E. coli *[21].

Limitations

The present study was conducted with a very small sample size, and only the antibiotic sensitivity and synergistic activity of E. coli organisms were observed. Therefore, future studies with larger sample sizes that include antibiotic sensitivity and synergistic activity of other organisms can be considered for future scope. Furthermore, future studies highlighting the individual effect of guava leaf extract can be focused on. Additionally, the antibiotic-plant extract synergy mechanism is still unknown. However, advancement in this field can lead to the use of a combination of extracts for the management of diseases, overcoming drug-resistant pathogens, and reducing the use of antibiotics and adverse effects caused by them.

## Conclusions

This study demonstrated the antimicrobial potential of Psidium guajava leaf extract by using distilled water as an aqueous solvent. The results reported that the flavonoid compounds and their derivatives in guava plant leaf extract can inhibit the growth of bacteria, and that the cumulative effect of guava leaf extract and antibiotics has a synergistic effect on uropathogenic E. coli. In the present study, the highest sensitivity of antibiotics with guava leaf extract was demonstrated by ofloxacin (100%). Therefore, patients suffering from UTI can be encouraged to consume guava fruit or guava juice as an adjunct to antibiotic treatment. Furthermore, in vivo studies might help to determine whether the consumption of guava juice or extract can increase the effectiveness of antibiotics and whether it can help to avoid the use of expensive, stronger, and toxic antibiotics for UTI treatment.
